# The Role of the Local Bone Marrow Renin-Angiotensin System in Multiple Myeloma

**DOI:** 10.4274/tjh.galenos.2019.2018.0420

**Published:** 2019-08-02

**Authors:** Bülent Saka, Müge Sayitoğlu, Zülal İstemihan, M. Akif Karan, Nilgün Erten, Öner Doğan, Uğur Özbek, Sema Genç, Cemil Taşçıoğlu, Sevgi Kalayoğlu-Beşışık

**Affiliations:** 1 İstanbul University, İstanbul Faculty of Medicine, Department of Internal Medicine, İstanbul, Turkey; 2 İstanbul University, Aziz Sancar Institute of Experimental Research, Department of Genetics, İstanbul, Turkey; 3 İstanbul University, İstanbul Faculty of Medicine, Department of Pathology, İstanbul, Turkey; 4 İstanbul University, İstanbul Faculty of Medicine, Department of Biochemistry, İstanbul, Turkey

**Keywords:** Multiple myeloma, Renin-angiotensin system, Angiotensin type 1a receptor

## Abstract

**Objective:**

Angiotensin II promotes growth and angiogenesis via type 1 receptors (AGTR1) in certain tumors. In this study, we examine the bone marrow AGTR1 expression in multiple myeloma (MM) and its relationship with the regulation of angiogenesis and prognostic factors.

**Materials and Methods:**

Bone marrow *AGTR1* mRNA levels of 39 MM patients and 15 healthy controls were analyzed with quantitative RT-PCR. Immunohistochemical staining of the tissue vascular endothelial growth factor (VEGF), CD34, and factor VIIIrAg (fVIIIrAg) was used to assess bone marrow angiogenesis.

**Results:**

Bone marrow samples of the patients showed increased VEGF, fVIIIrAg, and CD34 staining and higher *AGTR1* expression levels when compared to controls. Patients with severe-diffuse bone marrow infiltration showed higher bone marrow VEGF, fVIIIrAg, CD34, and *AGTR1* mRNA levels when compared to other patients.

**Conclusion:**

*AGTR1* expression was found positively correlated with plasma β2-microglobulin level and patients with increased AGTR1 expression showed increased bone marrow CD34 levels.

## Abstract (tr)

**Amaç:**

Anjiyotensin II, bazı tümörlerde tip 1 reseptörleri (AGTR1) yoluyla bir büyüme promotörü veya anjiyojenik faktör olarak görev yapar. Bu çalışmada, multipl myelomda (MM) kemik iliği AGTR1 ekspresyonunu ve bunun anjiyogenez ve prognostik faktörlerin düzenlenmesi ile ilişkisini incelemekteyiz.

**Gereç ve Yöntemler:**

Otuz dokuz MM hastası ve 15 sağlıklı kontrolde, kemik iliği *AGTR1* mRNA düzeyleri kantitatif RT-PCR ile analiz edildi. Kemik iliği anjiogenezi, doku vasküler endotel büyüme faktörü (VEGF), CD34 ve faktör VIIIrAg’nın (fVIIIrAg) immünohistokimyasal ölçümü ile değerlendirildi.

**Bulgular:**

Hastalar kontrollerle karşılaştırıldığında daha yüksek kemik iliği VEGF, fVIIIrAg, CD34 ve *AGTR1* ekspresyon seviyelerine sahipti ve şiddetli yaygın kemik iliği infiltrasyonu olan hastalar diğer hastalara göre daha yüksek kemik iliği VEGF, fVIIIrAg, CD34 ve *AGTR1* mRNA seviyeleri gösterdi.

**Sonuç:**

*AGTR1* ekspresyonu, plazma β2-mikroglobulin seviyesi ile korele bulundu ve artan AGTR1 ekspresyonu olan hastalarda kemik iliği CD34 seviyelerinde artış görüldü.

## Introduction

Side effects of angiotensin-converting enzyme inhibitors (ACEIs) such as anemia and leukopenia indicate inhibitory effects of these drugs on normal bone marrow hematopoiesis. With previous data on locally acting growth factor-like effects of angiotensin II (ATII), Haznedaroglu et al. [1] and Haznedaroglu and Ozturk [2] first mentioned a local renin-angiotensin system (RAS) in the bone marrow. The possible role of the bone marrow RAS was later reported in acute myeloid leukemia [3,4]. Abali et al. [5] showed increased bone marrow angiotensin-converting enzyme (ACE) levels compared to serum ACE in acute leukemia.

ATII was also related to angiogenesis, which could be inhibited with ACEI and ATII type 1a receptor (AGTR1) antagonists [6,7,8]. It can induce neovascularization due to increased expression of different growth factors (angiopoietin 2, vascular endothelial growth factor (VEGF), fibroblast growth factor, platelet-derived growth factor, transforming growth factor beta, and epidermal growth factor), nitric oxide synthase, and metalloproteinases [9]. Tamarat et al. [6] showed increased neovascularization with subcutaneous ATII injection in a rat model, which was found to be well correlated with serum VEGF and endothelial nitric oxide levels. AGTR1 antagonists and VEGF neutralizing antibodies completely prevented the ATII-induced angiogenesis. In another study, ATII was related to tumoral enlargement by inducing angiogenesis and malignant cell proliferation via *AGTR1*, while ACEI was shown to decrease cancer risk [8]. Egami et al. [10] compared rats with Agtr1+ and Agtr1- malignant melanoma and found decreased tumor angiogenesis and doubling time in Agtr1- rats, which resulted in increased survival rate. *AGTR1* antagonists showed suppressed tumor growth in Agtr1+ rats.

The interaction between malignant plasma cells and the bone marrow microenvironment is important in the etiopathogenesis of multiple myeloma (MM). Increased angiogenesis was shown in the bone marrow microenvironment, which was related to disease progression, resistance to treatment, and worse prognosis [11]. Tumor growth and angiogenesis may result from various cytokines and factors. VEGF is the best characterized pro-angiogenic factor produced by myeloma cells. It also stimulates stromal cells to produce interleukin-6, which is a potent myeloma growth factor [12,13].

The aim of this study was to find out any possible relationship between local bone marrow RAS activity and MM. Bone marrow RAS activities of patients were compared with their disease activity and bone marrow angiogenesis.

## Materials and Methods

### Patients and Controls

**Figure 1:**
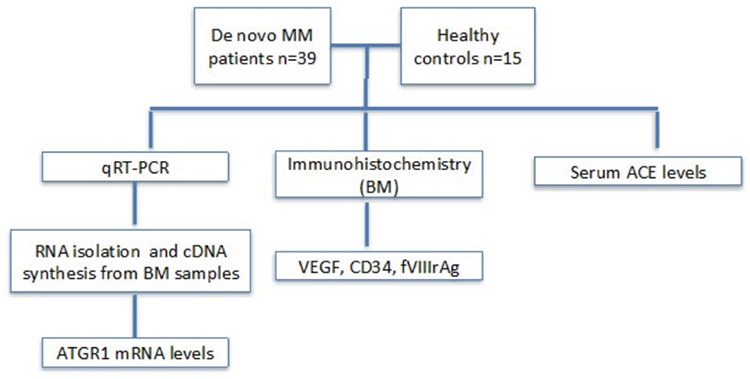
Flowchart of the study. AGTR1 mRNA: Angiotensin II type 1a receptor mRNA relative expression level, VEGF: vascular endothelial growth factor, MM: multiple myeloma, qRT-PCR: quantitative real-time polymerase chain reaction, ACE: angiotensin-converting enzyme.

De novo MM patients (n=39) without any previous treatment were enrolled in the study group. The control group (n=15) included healthy bone marrow donors and those with normal bone marrow histology who were examined clinically for any other reason (Figure 1). Patients and controls taking drugs with possible effects on the RAS were excluded (ACE inhibitors, *AGTR1* antagonists, beta blockers, spironolactone). Patients and controls with acute and/or chronic infectious diseases, inflammatory rheumatoid diseases, and any other cancer were also excluded. This study was approved by the Ethics Committee of İstanbul University İstanbul Medical Faculty (reference number 2008/305). Every patient included in the study provided signed informed consent.

### RNA Isolation and cDNA Synthesis

Bone marrow samples were collected in 2-mL ethylene diamine tetraacetic acid tubes. Total RNA was isolated from white blood cells (QIAGEN, Germany). RNA quality and quantity were measured by spectrophotometer (ND-1000, NanoDrop Technologies, Inc., USA), and 1 µg of total RNA was used. Random primers (20 µM, Roche Diagnostics, Germany), 10 mM dNTP set (Fermentas UAB, Lithuania), RiboLock RNase Inhibitor (20 U/µL, Fermentas), and Moloney murine leukemia virus reverse transcriptase (200 U/µL, Fermentas) were used for cDNA synthesis. cDNA samples were stored at -20 °C.

### Quantitative Real-Time Polymerase Chain Reaction Analysis

Real-time quantitative PCR was performed with a LightCycler 480 instrument (Roche Applied Sciences, Germany) (Table 1). Real-time amplification was performed with LightCycler 480 Probe Master Mix (Roche) according to the manufacturer’s protocol. Real-time amplification was performed with a final reaction mixture of 20 µL containing 5 µM of each primer, 0.5 µM of each probe, LightCycler 480 Probe Master Mix, and 100 ng/µL of cDNA. The three most stable genes *(B-ACTIN, CYCLOPHILIN A,* and *ABL)* were selected for normalization by geNorm software V3.4 (University of Liege, Belgium) (Table 1). Each sample was studied in duplicate and all runs were repeated twice. The PCR protocol was as follows: initial denaturation at 95 °C for 7 min, and amplification for 5 s at 95 °C, 10 s at 60 °C, and 10 s at 72 °C for 45 cycles. The ΔΔCt method was used to calculate relative expressions [14].

### Immunohistochemical Studies

**Figure 2:**
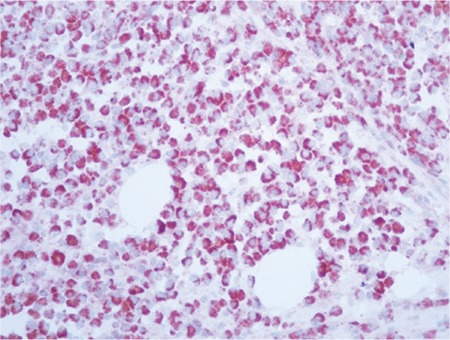
Bone marrow anti-vascular endothelial growth factor antibody, AEC chromogen, 400^x^.

**Figure 3:**
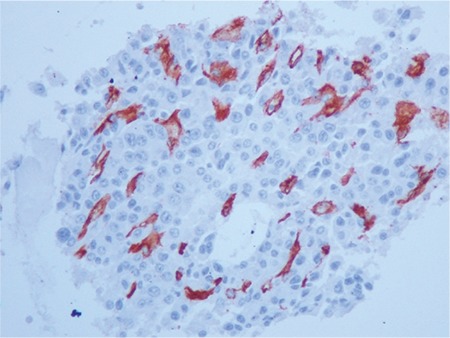
Bone marrow anti-CD34 antibody, AEC chromogen, 400^x^.

Bone marrow angiogenesis was evaluated with the immunohistochemical measurement of tissue VEGF (Figure 2), CD34 (Figure 3), and factor VIIIrAg (fVIIIrAg) indexes.

Bone marrow biopsy samples were fixed in formalin (10%) and then embedded into tissue paraffin blocks. After staining with hematoxylin and eosin, they were examined under a microscope. Tissue samples were incubated with anti-VEGF mouse monoclonal antibodies (clone G153-694) at 2 µg antibody/mL dilution. Immunocytochemical streptavidin-biotin peroxidase complex was used in the next stage, followed by diaminobenzidine chromogen for visualization of peroxidase reaction. Immunohistochemical staining activity was estimated semiquantitatively by using the immunoreactive score [15]. Scores were given to intensity of the reaction (stain) (0 to 3) and percentage of the cells with positive reaction (0 to 4). The final score was then obtained with the multiplication of both scores (0 to 12) (Table 2).

Staining of vascular endothelial cells with anti-CD34 murine monoclonal antibodies (Clone QBEnd/10, NeoMarkers, USA) at 1/100 dilution was also used to show bone marrow angiogenesis as described by Perez-Atayde et al. [16]. Bone marrow biopsy samples were examined at 80x magnification and five distinct fields were selected for evaluation. Mean number of CD34-stained vascular structures was defined as number of vessels/mm2. Factor VIIIrAg was also used to demonstrate bone marrow angiogenesis (Table 3).

Serum ACE levels of both groups were measured by sandwich ELISA method. One microplate was coated with an ACE-specific monoclonal antibody. Standards and serum samples were put in small Eppendorf tubes and ACE was bound with immobilized antibodies. After unbound materials were washed out of the wells, ACE-specific enzyme-linked polyclonal antibody was added to the tubes. Later, unbound antibody-enzyme particles were washed out, followed by the addition of substrate solution to samples. Color changes that occurred in the Eppendorf tubes were parallel to the ACE levels.

### Statistical Analysis

SPSS 15.0 was used to analyze data. Continuous variables were described with the use of statistical characteristics (means, standard deviations, median). Discrete variables were described as counts and percentages. The Kolmogorov-Smirnov test was used to analyze distribution of the variables. Independent samples t-tests, Mann-Whitney U tests, and Pearson correlation analyses were used during evaluation of the results. A value of p≤0.05 was considered statistically significant.

## Results

### AGTR1 mRNA Expression in MM Cases and Controls

**Figure 4:**
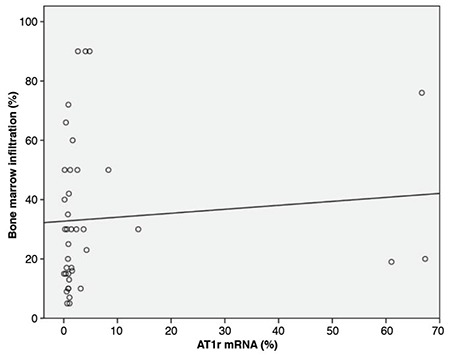
Correlation of the bone marrow infiltration ratio and *AGTR1* mRNA expression of the patients. AGTR1 mRNA: Angiotensin II type 1a receptor mRNA relative expression level.

**Figure 5:**
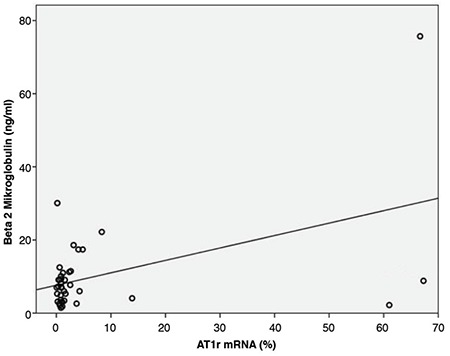
Correlation of the bone marrow *AGTR1* mRNA expression and serum β2-microglobulin levels of the patients. AGTR1 mRNA: Angiotensin II type 1a receptor mRNA relative expression level.

Thirty-nine MM patients (male/female: 20/19) were enrolled in the study. The control group included five healthy bone marrow donors and 10 people with normal cellular bone marrow biopsies who were examined for any other reason (male/female: 8/7). Mean ages of the patients and controls were 63±10 (minimum-maximum: 44-81) and 49±14 (minimum-maximum: 27-80) years, respectively. Clinical characteristics of the patients are given in Table 4. Bone marrow VEGF, CD34, and fVIIIrAg and *AGTR1* mRNA expression levels of the patients are given in Table 5. MM patients had higher bone marrow VEGF, CD34, and fVIIIrAg levels and increased *AGTR1* mRNA expression levels when compared to controls (Table 6). Patients with severe-diffuse bone marrow infiltration patterns showed higher bone marrow VEGF, CD34, and fVIIIrAg levels and higher bone marrow *AGTR1* mRNA expression when compared to others with mild-patchy infiltration patterns (Table 7; Figure 4). Plasma β2-microglobulin (B2M) concentrations of the patients were found to be well correlated with their bone marrow *AGTR1* mRNA expression levels (Figure 5; p=0.002). No association was found between disease stage and bone marrow *AGTR1* mRNA expression (p=0.760). Serum ACE levels of MM patients did not show any significant difference when compared to the control group (Student’s t-test).

Patients with higher *AGTR1* mRNA expression showed increased bone marrow CD34 (p=0.011, Student’s t-test). Similarly, patients with higher *AGTR1* mRNA expression showed increased bone marrow VEGF and fVIIIrAg indexes, although these did not reach statistical significance (VEGF: p=0.088, Mann-Whitney U test, fVIIIrAg: p=0.345, Student’s t-test).

## Discussion

The RAS has attracted attention because of its physiological and therapeutic potential. An extensive transcriptomic meta-analysis showed the expression patterns of RAS members in normal human tissues, including hematopoietic cells and bone marrow stem cells. AGT ligand was determined to be expressed in almost all tissue types, indicating its physiological importance. Bone marrow-derived cells have prominent expression of classical systemic RAS participants *(AGT-REN-ACE-AGTR1)* and they have almost the same expression patterns, indicating that transcriptional coordination may be preserved during cell lineage [17].

The RAS plays a role in hematopoietic stem cell plasticity. There is increasing evidence that the deregulated local bone marrow RAS could play a role in malignant transformation by increasing cellular proliferation and differentiation. ACE induces bone marrow stem cells to enter the S-phase through increasing hydrolysis of acetyl-N-Ser-Asp-Lys-Pro (AcSDKP), which inhibits the proliferation of bone marrow stem cells [18,19]. Conversely, ACEIs increase plasma AcSDKP and downregulate hematopoiesis [20]. ACEI treatment significantly decreased the hematocrit level of a patient with polycythemia vera [21].

Wulf et al. [3,4] showed renin-like activity in leukemic blast cells of a patient and isolated renin-like peptide from myeloblasts. Abali et al. [5] compared serum and bone marrow ACE concentrations of newly diagnosed acute leukemia patients and found significant increase in the latter. Serum ACE levels were found correlated with bone marrow infiltration rate and the number of blasts in the peripheral blood. RAS members’ expressions were detected in different myeloid blast cells [22,23]. RAS and NOTCH pathways are in communication, and the *RBP-J *gene (recombination signal binding protein for immunoglobulin kappa J region) is an important transcriptional regulator of the NOTCH pathway. An *RBP-J*-deleted mouse model showed *Ren* expression leading to leukemogenesis in B-cell progenitors. Moreover, there are limited data showing *RBP-J* gene mutations in leukemia patients [24,25]. Serum ACE was found increased in MM patients and local RAS components were also found in the following studies [26,27].

ATII plays a fundamental role in controlling cardiovascular function and renal homeostasis. It has many physiologic effects other than regulating vascular tone, such as hormone secretion, tissue growth, and neural activity. It has four receptors. AGTR1 stimulation activates intracellular pathways that finally lead to vasoconstriction, inflammation, and proliferation [9]. Like other cytokines, ATII was shown to use the JAK-STAT pathway (JAnus or Just Another Kinase-Signal Transducers and Activators of Transcription) in the regulation of hematopoiesis [28]. Gomez et al. [29] revealed the ability of rat leukocytes to produce angiotensinogen and ATII, and Crabos et al. [30] found Agtr1 on thrombocytes. Rodgers et al. [31] showed Agtr1 on CD34+CD38- and CD34+CD38+ cells, lymphocytes, and bone marrow stromal cells, and they reported increased bone marrow stem cell proliferation with ATII that was inhibited with the AGTR1 antagonist losartan. Mrug et al. [32] reported similar effects of the local bone marrow RAS on the erythroid cell lineage. Jokubaitis et al. [33] identified a 160-kDa cell surface glycoprotein, BB9, which is found on hematopoietic stem cells (HSCs) throughout hematopoietic development, even at the earliest definitive phases. They demonstrated that BB9 monoclonal Ab identifies the somatic form of angiotensin-converting enzyme (ACE/CD143), which suggested its expression by HSCs from primitive phases to adulthood. ACE/CD143 may thus play a role in the regulation of hematopoietic cells.

According to our results, the bone marrow *AGTR1* expression of our patients showed positive correlation with their bone marrow infiltration pattern and serum B2M levels. Serum B2M level and the morphology of myeloma cells are reliable prognostic factors in MM. Moreover, serum B2M was found as the most important parameter in predicting high-risk patients [34,35]. The positive correlation between bone marrow *AGTR1* mRNA levels, bone marrow morphology, and plasma B2M showed that bone marrow *AGTR1* expression can give information about prognosis in MM.

Increased bone marrow VEGF, CD34, and fVIIIrAg indexes in our patients reflected neovascularization. Advancing age is associated with the development of vascular endothelial dysfunction. Vascular oxidative stress increases with age as a consequence of greater production of reactive oxygen species (e.g., superoxide) without a compensatory increase in antioxidant defenses. In our study, the control group seemed to be younger than the MM group (range: 27-80 years), but was within the age range at which MM develops. Patients with higher *AGTR1* expression showed increased bone marrow CD34 index. In our opinion, the statistically nonsignificant increase in the bone marrow VEGF and fVIIIrAg indexes of these patients was related to the number of subjects enrolled in the study.

Bone marrow *AGTR1* mRNA expression was 2%-4% in 5 patients and >4% in 8 patients. Three patients had extremely high levels of expression (67.29%, 66.67%, and 61.02%) when compared to others. The first patient was a 69-year-old man with IgG kappa light chain MM of stage 3. He had 30% bone marrow infiltration and his serum monoclonal band, lactate dehydrogenase (LDH), and B2M were 5.28 g/dL, 412 IU/L, and 8.83 ng/mL, respectively. The second patient was a 51-year-old woman with kappa light chain secreting disease of stage 3. Her laboratory analysis revealed overt disease activity (76% bone marrow infiltration, serum monoclonal band 6.9 g/dL, LDH 696 IU/L, and B2M 75.76 ng/mL). The third patient was a 67-year-old woman with IgA lambda light chain secreting disease of stage 2. She had mild disease activity at diagnosis (21% bone marrow infiltration, serum monoclonal band 1.94 g/dL, LDH 264 IU/L, and B2M 2.24 ng/mL). Bone marrow VEGF, CD34, and fVIIIrAg indexes of these three patients were also found to be significantly increased.

Only 3 control subjects showed 2%-4% bone marrow *AGTR1* mRNA expression. Two of them were under medical examination for another reason and showed normocellular bone marrow histology, and the third one was a healthy bone marrow donor. Moreover, they had relatively low levels of bone marrow VEGF, CD34, and fVIIIrAg indexes when compared with MM patients.

A clinically relevant aspect of the interactions of MM plasma cells in the bone marrow microenvironment is neovascularization, a constant hallmark of disease progression. Myeloma plasma cells also induce angiogenesis indirectly via recruitment and activation of stromal inflammatory cells (i.e. macrophages and mast cells) to secrete their own angiogenic factors. RAS signaling pathway mutations have been reported in newly diagnosed MM cases and even more so in relapsed/refractory MM [36,37], which could correlate with ACE expression level. Both findings may encourage the use of ACEIs or mitogen-activated protein kinase inhibitors in MM.

### Study Limitations

The limitation of this study was the low number of MM patients enrolled, which was caused by the planned time schedule and the difficulty of finding de novo myeloma patients.

## Conclusion

Bone marrow *AGTR1* expression can give information about bone marrow morphology and can predict disease progression in MM. Further studies are needed to ascertain such an association.

## Figures and Tables

**Table 1:**
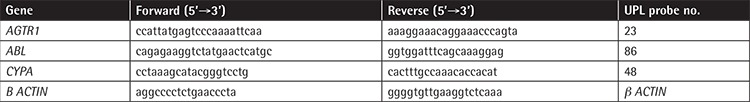
Primers and probes used in quantitative real-time polymerase chain reaction.

**Table 2:**
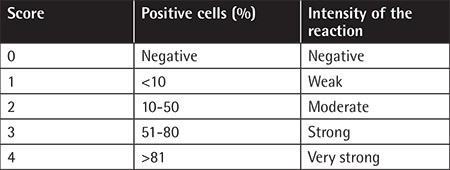
Estimation of the bone marrow vascular endothelial growth factor index with immunoreactive score.

**Table 3:**
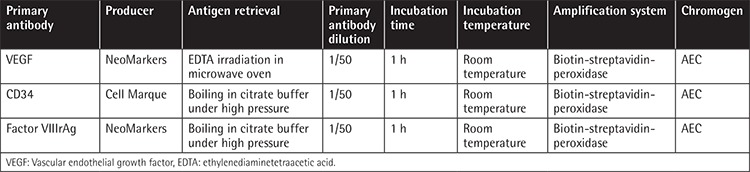
Bone marrow immunohistochemical studies of angiogenesis.

**Table 4:**
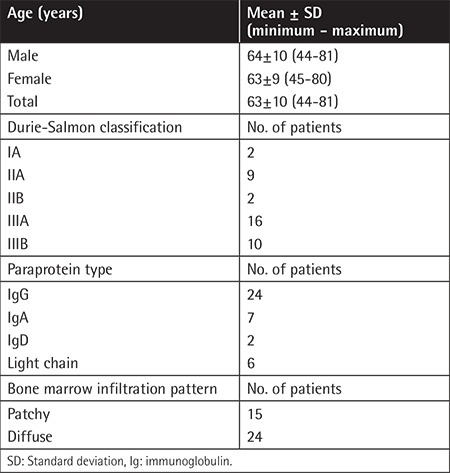
Clinical characteristics of the patients.

**Table 5:**
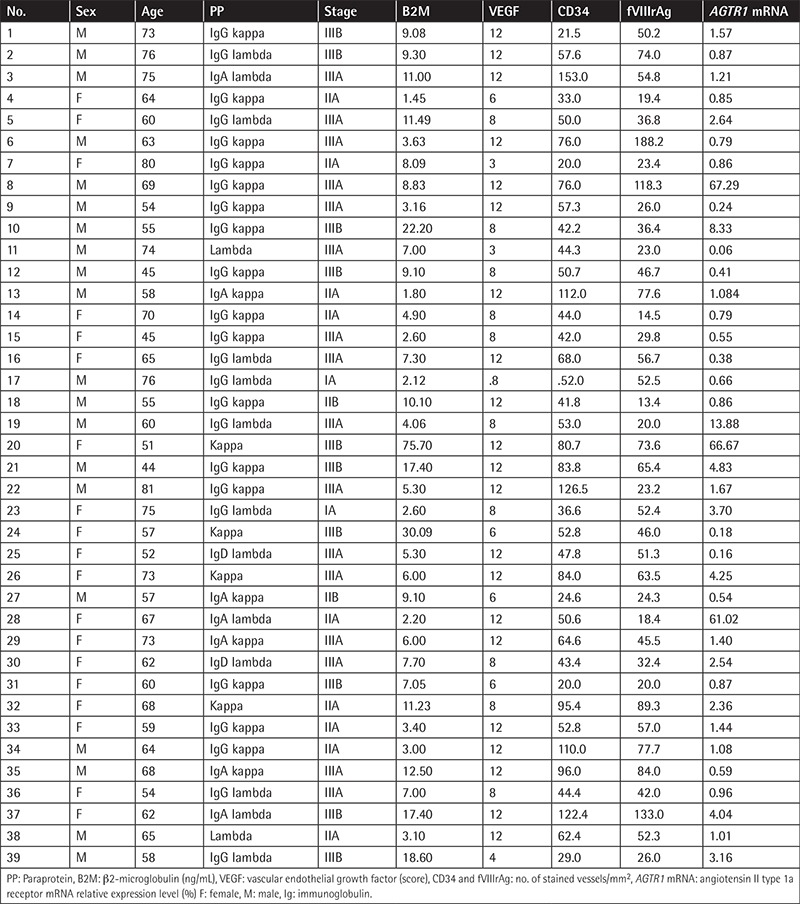
Patient group characteristics, bone marrow proangiogenic factors, and *AGTR1* mRNA relative expression.

**Table 6:**
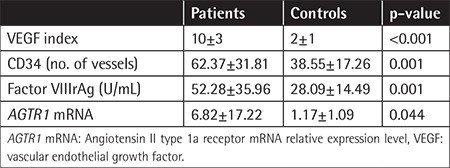
Angiogenesis factors and *AGTR1* mRNA expression levels of the patients and controls.

**Table 7:**
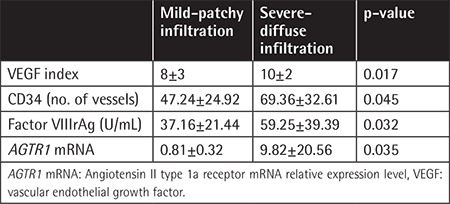
Angiogenesis factors and *AGTR1* mRNA expression levels according to bone marrow infiltration pattern of the patients.
